# Unusual Long-Term Survival in an Adult Patient With Langerhans Cell Histiocytosis and Central Nervous System Involvement: A Case Report

**DOI:** 10.1155/crh/6031427

**Published:** 2025-05-16

**Authors:** María José Miranda Pallarés, Tomás Álvaro Naranjo, Anna Vidal Rodriguez, Francisca Martínez Madueño

**Affiliations:** ^1^Medical Oncology Unit, Institut d'Oncologia de la Catalunya Sud (IOCS), Hospital Universitari Sant Joan de Reus, Reus 43204, Catalonia, Spain; ^2^Translational, Epidemiological and Clinical Oncological Research Group (GIOTEC), Institut d'Investigació Sanitària Pere Virgili (IISPV), Reus 43204, Catalonia, Spain; ^3^Service of Pathology, Hospital de Tortosa Verge de la Cinta-ICS Ebre, Tortosa, Tarragona, Spain; ^4^Departament de Ciències Mèdiques Bàsiques, Universitat Rovira i Virgili, Reus 43204, Catalonia, Spain

**Keywords:** adult, BRAF mutation, dendritic cells, diabetes insipidus, Langerhans cells, Langerhans cell histiocytosis, lymphadenopathy, MAP2K1 mutations

## Abstract

Langerhans cell histiocytosis (LCH) in adults is a rare condition. The disease presents with focal or multifocal involvement of a single organ/system or focal or disseminated multisystem disease. Involvement of the central nervous system (CNS) is also infrequent, with diabetes insipidus as a common manifestation of posterior pituitary lesions. Biopsy-proven diagnosis with the observation of characteristic Langerhans cells infiltrate with positive immunohistochemistry of S100 protein, CD1a, and CD68 cells. The patient presented generalized lymphadenopathy and was diagnosed with low-risk single-system LCH-based distinctive pathological findings in a lymph node biopsy. During the disease, CNS involvement was documented and the patient received different sequential therapeutic schemes, achieving complete remission that has been maintained for 8 years. The prolonged duration of remission with disappearance of lymphadenopathy and CNS lesions in an adult patient with LCH is an unusual clinical observation.

## 1. Introduction

Langerhans cell histiocytosis (LCH) is a rare systemic hematologic disorder caused by clonal expansion of myeloid precursors that differentiate into CD1a^+^/CD207^+^ dendritic cells in lesions that leads to a spectrum of organ involvement and dysfunction [1, 2]. The disease is more common in children than in adults, and in men than in women. The incidence of LCH in adults is poorly defined. According to the Surveillance, Epidemiology, and End Results (SEER) Program database, 59 cases of adult LCH have been collected between 1973 and 2009, with an overall incidence of 0.07 per 1,000,000 individuals (95% confidence interval [CI] 0.05–0.10) [3]. In the 2016 Histiocyte Society classification [4], LCH was reclassified as clonal inflammatory myeloid neoplasm harboring genetic alterations leading to aberrant oncological signaling [5, 6], with *BRAF V600E* and *MAP2K1* gain-of-function mutations found in more than 70% of LCH patients [7].

Clinically, LCH is a heterogeneous disease that may involve a single organ (single-system LCH) (unifocal or multifocal) or multiple organs (multisystem LCH). It is important to identify involvement of “risk organs” (hematopoietic system, liver, and spleen) or “critical organs” (central nervous system [CNS] and lung) [4, 8]. The features of the disease remain poorly defined in adults. In a review of 274 patients (18 years or older) from the International Histiocyte Society Registry [9], single-system LCH was found in 86 patients (31.4%) and multisystem in 188 (68.6%), with lung (58.4%), bone (57.3%), and skin (36.9%) as the most frequent sites of involvement. In a 50-year experience at the Mayo Clinic with 314 patients (mean age 24.5 years), multisystem disease was diagnosed in 30.6% of patients, with bone involvement in 36.3% of cases, pulmonary in 17.7%, and mucocutaneous in 4.4% [10].

Lesions in the CNS in adult patients with LCH are uncommon. Apart from infiltration of the pituitary gland causing diabetes insipidus (DI), other forms of CNS involvement and neuroradiological abnormalities, with or without neurological dysfunction developed during the disease, varied widely [11, 12]. The rarity and insufficient knowledge of CNS involvement in adults with LCH may result in missed or delayed diagnosis. Patients with CNS disease are difficult to manage and frequently require different salvage regimens [12–14]. However, most of them relapse upon discontinuation of therapy, and the outcome is poor, especially in multifocal involvement and in the presence of neurodegenerative syndrome [15, 16]. We here report a case of unusual long-term survival in a young man with LCH and CNS disease.

## 2. Case Report

A 21-year-old Caucasian man, with a history of asthma and who was a current smoker, presented in February 2011 with an episode of fever up to 38°C in the evenings that lasted several days without other associated symptoms. The chest X-rays and standard hematological, biochemical, and urinalysis were unrevealing except for an increase in serum C-reactive protein (CRP) of 22 mg/L. A thoracoabdominal computed tomography (CT) scan revealed multiple lymph nodes in the interaortocaval region, the external and internal common iliac chains, the bilateral internal obturators, and the right common femoral chain. Notably, the largest nodes measured 35 mm and 45 mm on the left and right internal obturator chains, respectively, and there was a 25-mm node in the right common femoral region.

**Figure 1:**
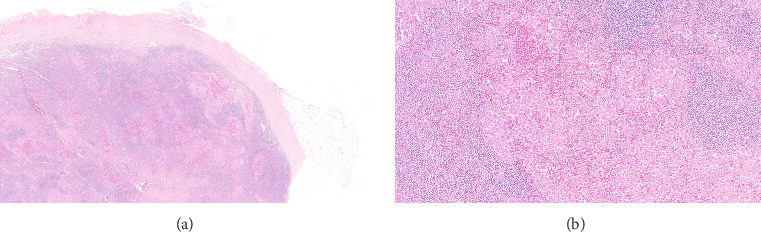
(a, b) Microscopic examination at low and medium magnification shows architectural disruption, presence of mixed cellular nests with a predominance of epithelioid histiocytes mixed with lymphocytes and numerous eosinophils. Cytologically, nuclear grooves, pale cytoplasm, and absence of mitosis are observed (hematoxylin and eosin staining).

An excisional biopsy of a right inguinal adenopathy was performed. Details of microscopic examination and immunohistochemical staining are shown in Figures 1(a) and 1(b). Microscopic examination at low magnification showed architectural alteration of the lymph node parenchyma, with preservation of some follicular centers, and sinusoidal infiltration forming nests by the accumulation of large epithelioid cells mixed with lymphocytes and numerous eosinophils. Fibrosis and perinodal infiltration were also present. Foci of necrosis of diffuse infiltration were not observed. At medium and high magnification, large cells were identified, occasionally multinucleated, with fine chromatin, minimal atypia, no prominent nucleolus, nuclear grooves and indentations, and a large amount of pale cytoplasm. No mitoses were found nor were there Sternberg-like cells. No ultrastructural study was performed. The immunohistochemical analysis revealed intense and diffuse staining for CD1a, CD4, S100 protein, and CD68 markers, with moderate staining for CD45, and negative staining for other B- and T-cell line markers, as well as for CD21, CD30, and HMB45.

**Figure 2:**
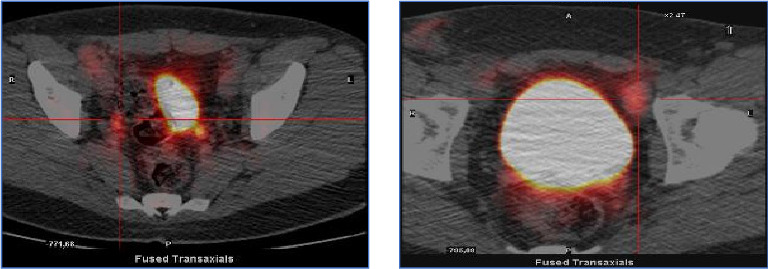
Initial positron emission tomography (PET).

Next‐generation sequencing molecular analysis was negative for *BRAF* and *c-kit* mutations. A positron emission tomography (PET) confirmed generalized lymphadenopathy (SUV_max_ 4) and small doubtful bilateral supraclavicular adenopathy of brown fat (Figure 2).

Based on lymphadenopathy in the absence of involvement of risk organs, the patient was diagnosed with low-risk single system LHC and received chemotherapy with vinblastine, 6 mg/m^2^/day intravenously (i.v.) every week for 7 weeks and oral prednisone 40 mg/m^2^ for 4 weeks in the induction phase, followed by maintenance with vinblastine and prednisone every 21 days until completing 6 months of treatment. A PET scan after the induction phase showed a complete metabolic response at the lymph node level.

The treatment was definitively ended in September 2011.

**Figure 3:**
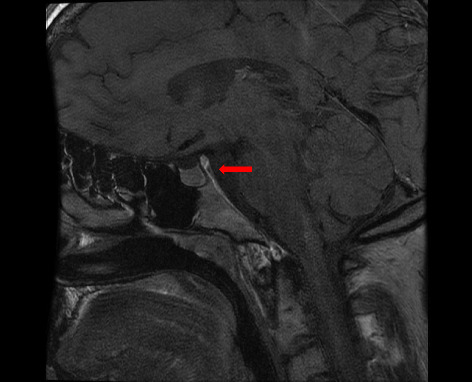
Brain magnetic resonance imaging (MRI). Sagittal view shows absence of normal hyperintense T1 signal in the neurohypophysis in the posterior intrasellar localization (arrow) in relation to diabetes insipidus.

The patient remained asymptomatic, and CT controls did not reveal the relapses of the disease until July 2013, the time at which he presented clinical symptoms of polydipsia and polyuria. The brain magnetic resonance imaging (MRI) scan showed the absence of the normal hyperintense signal in T1 of the neurohypophysis in the posterior intrasellar location (Figure 3) compatible with the onset of DI. Synchronously, a PET scan revealed a relapse of lymphadenopathy, with predominant uptake of bilateral cervical and inguinal lymph nodes (SUV_max_ 6).

Symptoms of DI were controlled with desmopressin treatment. Histological diagnosis was attempted without success; but given the close relationship of DI with histiocytosis, relapse of LCH was assumed, which occurred almost 2 years after the end of the initial treatment.

In August 2013, the patient was treated again with vinblastine and prednisone. After induction, a complete response was achieved, although dose adjustment was required due to neurotoxicity in the upper extremities.

During maintenance treatment, respiratory symptoms worsened and a fiberoptic bronchoscopy and a high-resolution CT scan were performed. In the bronchoalveolar lavage fluid, 2% of CD1a cells were detected, but given the small percentage of cells and the fact that the pulmonary parenchyma was unaffected, the respiratory condition was considered unrelated to LCH.

**Figure 4:**
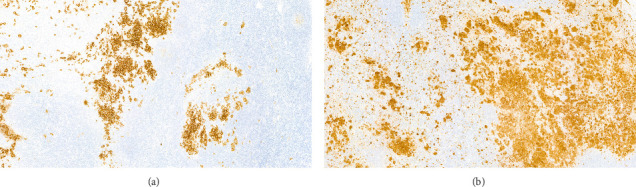
(a) CD1 staining shows intense and diffuse positivity over the cellularity with Langerhans cell morphology. (b) Immunohistochemistry for S100 protein shows intense positivity over the infiltrate.

The patient refused to continue chemotherapy and stopped treatment in June 2014, 10 months after starting chemotherapy. In July 2014, one month after stopping the treatment, he was attended to the emergency department because of perioral paresthesia, headache, nausea, and vomiting. A PET/CT scan showed a hyperintense nodular mass in the subputaminal nucleus on the left side, which has not been present on previous examinations, together with small bilateral laterocervical lymph (12-14 mm in diameter) (SUV_max_ 6–9). A cranial MRI scan showed osteolytic involvement with soft tissue components on the lateral wall of the right orbit, left posterior parietal leptomeningeal uptake, and another 4-mm left temporal-hippocampal intraparenchymal lesion. Three cervical lymph nodes were removed and the histopathological examination confirmed the presence of Langerhans cells with high expression of CD1A and S100 protein (Figures 4(a) and 4(b)). Lumbar puncture and analysis of the cerebrospinal fluid (CSF) were unrevealing.

In August 2014, the patient received treatment with cranial radiotherapy with a total of 36 Gy with an early clinical response and rapid disappearance of headache. Subsequently, the patient was treated with cladribine 5 mg/m^2^/day i.v. for 5 days every 21 days up to a total of six cycles. A control MRI scan performed 2 months after starting radiation therapy showed the disappearance of the intraparenchymal lesion and leptomeningeal enhancement with a decrease in the lesion of the right orbit, and the PET/CT scan after completing two cycles of cladribine showed a complete metabolic response.

**Figure 5:**
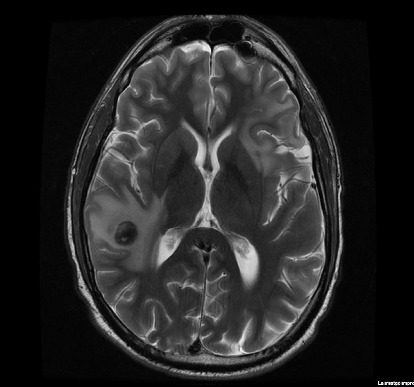
Brain MRI response. Axial view revealed the presence of intra-axial lesions, one at the right temporal region (with evident perilesional edema 15 mm in diameter) and another at the left frontal region in the cortico-subcortical area with marked perilesional edema. Lesions showed low signal intensity on T2-weighted images and FLAIR.

At the end of treatment, the PET/CT scan disclosed 16-mm intraparenchymal lesions in the right temporal region, 11-mm left frontal, and 6-mm left parietal with moderate perilesional edema and slight mass effect, with stability of the lesion located and treated in the right orbit. Slightly enlarged lymphadenopathies reappeared with low SUV_max_ 3-4. The MRI images confirmed it (Figure 5). Treatment with cytosine arabinoside, 500 mg/m^2^, and vincristine 1.5 mg/m^2^ in 18-day cycles was initiated.

After 4 months of treatment, PET-CT and MRI scans showed the complete metabolic response of the lymph node involvement with the disappearance of intraparenchymal cerebral lesions and normal uptake of the lesion in the right orbit. However, the patient presented Grade 4 hematological toxicity despite dose adjustment and the administration of granulocyte-colony stimulating factors (G-CSF).

In January 2016, the patient had received up to seven cycles but due to the reappearance of supradiaphragmatic lymph nodes involvement with little uptake and hematological toxicity, the treatment was switched to oral mercaptopurine, 100 mg/day in March 2016. A bone marrow study ruled out infiltration by Langerhans cells or other hematological abnormalities. After 6 months of oral treatment with mercaptopurine, active surveillance was decided considering the number of treatment lines administered and the fact that lymph node involvement remained stable (in size and low SUV_max_).

The reappearance of symptoms of polydipsia and polyuria was controlled with intranasal desmopressin, with the final resolution of symptomatology in January 2020.

At follow-up and after completing treatment with mercaptopurine, control MRI and CT examinations did not show signs of progression of CNS disease, and he remained in good condition with normal life activities in the last follow-up on June 2024.

A summary of salient features and treatment regimens is shown in Table 1.

## 3. Discussion

The patient here reported presented generalized lymphadenopathy and was diagnosed with low-risk single-system LCH based on distinctive pathological findings in a lymph node biopsy, characterized by proliferation of Langerhans cells with intense vimentin, S100 protein, CD1a, and CD68 expression. During the disease, CNS involvement was documented and the patient received different sequential therapeutic schemes, achieving complete remission that has been maintained for 8 years. The prolonged duration of remission with the disappearance of CNS lesions in an adult patient with LCH is an unusual clinical observation that has not been previously reported in the literature.

Adult-onset LCH is a rare condition. Also, insufficient knowledge of LCH in adults, especially CNS involvement in LCH, often results in missed and delayed diagnosis. Recently, Liu et al. [17] reported the case of a 35-year-old woman, who remained undiagnosed for 10 years, with neuroendocrine and neuropsychological disturbances and a mass lesion in the hypothalamic-pituitary region and drew attention to the awareness of CNS involvement given the myriad manifestations of LCH in peripheral organ/systems which may further complicate the diagnosis. In our patient, the diagnosis of LHC was promptly established through the indication of a lymph node biopsy to determine the etiology of his lymphadenopathy. The clinical presentation of LCH limited to the lymph nodes has been also reported in other clinical series including pediatric and adult patients [18, 19].

Treatment of LCH depends on the site and extension of the disease. In patients with isolated mild pulmonary LCH, smoking cessation is frequently the only therapeutic intervention, but systemic therapy is indicated in patients with multisystem and multifocal single-system disease. Unlike pediatric LCH, a first-line chemotherapy treatment has not been standardized for adult LCH patients. Vinblastine + steroids is an effective and well-tolerated first-line treatment for adult LCH, although a significant proportion of patients may experience reactivation during long-term follow-up. In a series of 35 adults with LHC treated with vinblastine + steroids as first-line chemotherapy (median duration of treatment 7.6 months) and followed for a median time of 83 months, 70% were responders and subsequent LCH reactivation occurred in 49% of cases, half of which were retreated with vinblastine [20]. In a retrospective chart review of 20 patients from the French LCH Study Group, vinblastine with or without steroids, concomitantly, complete and partial response was obtained in 15 patients, four had stable disease and one progressed [14]. Although vinblastine + prednisone is a reasonable alternative, the risk of relapse is high, so chemotherapy using either cladribine or cytarabine may be preferred because of relatively high overall response rates and the potential for long-term remissions with limited cycles of treatment [21].

In our patient, initial chemotherapy with vinblastine + prednisone achieved a complete response, but cladribine and cytosine arabinoside were subsequently administered for lymphadenopathy relapse and CNS lesions, which had to be discontinued and switch to oral mercaptopurine due to severe hematotoxicity.

Targeted therapy with BRAF and MEK inhibitors would not have been adequate in our patient as somatic mutations were not identified.

DI was another clinical manifestation of the patient. LCH exhibits a predilection for the hypothalamic-pituitary-axis, with DI being the most common endocrine consequence related to the disease, which may be before diagnosis, at the time of presentation, or develop at any time during the disease [22–24]. In pediatric LCH, older chronological age at diagnosis, low-risk clinical forms, and fewer reactivation episodes represent a subgroup of patients with a higher risk of developing anterior pituitary hormone deficiencies and DI [25]. In our patient, DI appeared at the initial manifestations of CNS involvement and lymphadenopathy relapse and was successfully controlled with desmopressin.

A neurodegenerative disease that occurs as a CNS manifestation in about 5%–10% of patients with LCH, especially in patients with multisystem forms, is a severe complication that may develop even years after assumed remission [26, 27]. Clinical symptoms with ataxia, dysarthria, dysmetria, and alterations in behavior and learning have been reported, as well as increased T2-weighted MRI signal in the dentate nucleus of the cerebellum, basal ganglia as characteristics of MRI findings in LCH associated with neurodegeneration [11]. So far, our patient has not presented this complication.

## 4. Conclusion

LCH in adults is a rare condition with a wide clinical spectrum, ranging from unifocal single organ/system involvement to disseminated multisystem disease. The lack of awareness of LCH by physicians may often lead to delayed diagnosis and misdiagnosis. The diagnostic clue is based on histopathological identification of characteristic Langerhans cell infiltration with positive vimentin, S100 protein, CD1a, and CD68 staining. Salient features of the patient reported here include generalized lymphadenopathy with CNS involvement; a clinical course characterized by remissions and relapses after different chemotherapies with vincristine + prednisone, cladribine, cytosine arabinoside, and mercaptopurine leading to cure; and the long-term period in good clinical condition, absence of symptoms, and maintaining a normal daily life.

## Figures and Tables

**Table 1:** Clinical features and successive treatment strategies in the present patient with Langerhans cell histiocytosis (LCH).

Date	Main condition	Treatment	Outcome
February, 2011	Lymphadenopathy Low-risk single-system LCH	Vinblastine and prednisone 6 cycles	Complete response
July, 2013	Lymphadenopathy relapse and diabetes insipidus	Retreatment with vinblastine and prednisone for 10 months	Complete response after induction
August, 2014	Lymphadenopathy relapse and CNS involvement	Brain radiotherapy and Cladribine, 6 cycles	Initial partial response but rapid CNS relapse
April, 2015	Progressive lymphadenopathy and hematotoxicity Grade 4	Cytosine arabinoside 7 cycles	Progressive disease lymph nodes
March, 2016	Treatment change due to hematotoxicity	Mercaptopurine 6 months	Complete response lymphadenopathies and CNS involvement
June, 2024	Asymptomatic Normal daily activities	None	Complete response lymphadenopathies and CNS involvement

Abbreviation: CNS, central nervous system.

## Data Availability

The histopathological, radioimaging, and laboratory data used to support the findings of this study are available from the corresponding author upon request.
